# Assisted Reproductive Technology and Risk of Childhood Cancer Among the Offspring of Parents With Infertility: Systematic Review and Meta-Analysis

**DOI:** 10.2196/65820

**Published:** 2025-03-12

**Authors:** Gao Song, Cai-qiong Zhang, Zhong-ping Bai, Rong Li, Meng-qun Cheng

**Affiliations:** 1 Department of Pharmacy, Puer People's Hospital, Puer, China; 2 Department of Reproductive Medicine, Puer People's Hospital, 44 Zhenxing Avenue, Puer, Yunnan, 665000, China, 86 18082997667, 86 2121114

**Keywords:** assisted reproductive technology, childhood cancer, infertility, subfertile, risks, systematic review

## Abstract

**Background:**

The relationship between assisted reproductive technology (ART) and childhood cancer risk has been widely debated. Previous meta-analyses did not adequately account for the impact of infertility, and this study addresses this gap.

**Objective:**

Our primary objective was to assess the relative risk (RR) of childhood cancer in infertile populations using ART versus non-ART offspring, with a secondary focus on comparing frozen embryo transfer (FET) and fresh embryo transfer (fresh-ET).

**Methods:**

A literature review was conducted through PubMed, Embase, Cochrane, and Web of Science, with a cutoff date of July 10, 2024. The study was registered with the International Platform of Registered Systematic Review and Meta-Analysis Protocols (INPLASY 202470119). Inclusion criteria were based on the PICOS (Population, Intervention, Comparison, Outcomes, and Study Design) framework: infertile or subfertile couples (population), ART interventions (in vitro fertilization [IVF], intracytoplasmic sperm injection [ICSI], FET, and fresh-ET), non-ART comparison, and childhood cancer risk outcomes. Data abstraction focused on the primary exposures (ART vs non-ART and FET vs fresh-ET) and outcomes (childhood cancer risk). The risk of bias was assessed using the Newcastle-Ottawa Quality Assessment Scale, and the evidence quality was evaluated with the Grading of Recommendations Assessment, Development, and Evaluation (GRADE). Pooled estimates and 95% CIs were calculated using random effects models.

**Results:**

A total of 18 studies were included, published between 2000 and 2024, consisting of 14 (78%) cohort studies and 4 (22%) case-control studies, all of which were of moderate to high quality. The cohort studies had follow-up periods ranging from 3 to 18 years. Compared with non-ART conception, ART conception was not significantly associated with an increased risk of childhood overall cancer (RR 0.95, 95% CI 0.71‐1.27; GRADE quality: low to moderate). Subgroup analyses of IVF (RR 0.86, 95% CI 0.59‐1.25), ICSI (RR 0.76, 95% CI 0.26‐2.2), FET (RR 0.98, 95% CI 0.54‐1.76), and fresh-ET (RR 0.75, 95% CI 0.49‐1.15) showed similar findings. No significant differences were found for specific childhood cancers, including leukemia (RR 0.99, 95% CI 0.79‐1.24), lymphoma (RR 1.22, 95% CI 0.64‐2.34), brain cancer (RR 1.22, 95% CI 0.73‐2.05), embryonal tumors (RR 1, 95% CI 0.63‐1.58), retinoblastoma (RR 1.3, 95% CI 0.73‐2.31), and neuroblastoma (RR 1.02, 95% CI 0.48‐2.16). Additionally, no significant difference was observed in a head-to-head comparison of FET versus fresh-ET (RR 0.99, 95% CI 0.86‐1.14; GRADE quality: moderate).

**Conclusions:**

In conclusion, this study found no significant difference in the risk of childhood cancer between offspring conceived through ART and those conceived through non-ART treatments (such as fertility drugs or intrauterine insemination) in infertile populations. While infertility treatments may elevate baseline risks, our findings suggest that whether individuals with infertility conceive using ART or non-ART methods, their offspring do not face a significantly higher risk of childhood cancer. Further research, especially comparing infertile populations who conceive naturally, is needed to better understand potential long-term health outcomes.

## Introduction

Over the last century, global fertility rates have significantly declined, and it is projected that by 2060, fertility will fall below replacement levels [1,2]. This trend is closely linked to an increase in infertility, which can be caused by factors such as ovulation disorders, tubal abnormalities, uterine issues, and sperm abnormalities [3]. Assisted reproductive technology (ART) has helped many infertile couples achieve parenthood. Since ART’s introduction in 1978, over 10 million children have been born using this technology [4], with approximately 1 million children conceived via ART each year. As ART usage increases, monitoring the long-term health risks associated with it, particularly childhood cancer, becomes crucial [5].

The relationship between ART and childhood cancer has been widely studied, but the results remain controversial due to inconsistent findings [6,7]. One of the key reasons for this inconsistency is the use of different reference groups. Few studies distinguish between children born to parents with infertility and those born to parents who conceived naturally [5,8]. It is essential to differentiate the effects of parental infertility from those of ART treatment, particularly given the challenge of small sample sizes in many studies. Furthermore, most studies are conducted within a single health care system or region, which limits their ability to fully assess cancer risk in offspring conceived through ART.

Previous reviews and meta-analyses have not adequately addressed infertility as a factor, possibly due to the limited availability of relevant studies [9-14]. However, recent large national cohort studies have compared offspring of parents with infertility with controls, and follow-up periods have extended beyond 10 years [15-22]. Given these advances, we conducted a systematic review and meta-analysis to assess the relative risk (RR) of childhood cancer in ART versus non-ART offspring in infertile populations and to compare frozen embryo transfer (FET) with fresh embryo transfer (fresh-ET). This study provides new insights into the relationship between ART modalities and pediatric cancer risk, which could help guide clinical ART fertility treatments.

## Methods

### Overview

This study was retrospectively registered with the International Platform of Registered Systematic Review and Meta-Analysis Protocols (INPLASY 202470119). The systematic review followed the PRISMA (Preferred Reporting Items for Systematic Reviews and Meta-Analyses) guidelines and included all published articles on ART exposure and childhood cancer risk in the offspring of parents with infertility [23].

### Search Strategy and Inclusion Criteria

We conducted a systematic literature search with a deadline of July 10, 2024, using PubMed, Embase, Cochrane, and Web of Science. The electronic search strategy was initially developed by the author (GS) and subsequently reviewed by the author with extensive search experience (MQC). We first tested the search by adapting it for each database and validating it against previously published meta-analyses on relevant topics to ensure the comprehensiveness of our approach. The validated search strategy was implemented simultaneously across each database on July 10, 2024, using the search terms “ART,” “children,” “cancer,” and “risk.” The detailed Boolean expressions of the search strategy are presented in Multimedia Appendix 1.

The inclusion criteria were constructed using the PICOS (Population, Intervention, Comparison, Outcomes, and Study Design) framework:

- Population: infertile or subfertile couples.
- Intervention: ART, including in vitro fertilization (IVF), intracytoplasmic sperm injection (ICSI), FET, and fresh-ET.
- Comparison: non-ART, defined as infertile or subfertile couples who did not conceive through ART but may have conceived naturally or with induced ovulation induction (OI) or intrauterine insemination (IUI).
- Outcomes: risk of childhood cancer, including overall childhood cancers and specific types such as leukemia, lymphomas, brain cancer, embryonal tumors, retinoblastoma, and neuroblastoma.
- Study design: randomized controlled trials and observational studies (eg, cohort or case-control studies).

Studies lacking sufficient data to calculate RR estimates and their 95% CIs were excluded. Additionally, conference abstracts, reviews, non-English articles, duplicate data, and non–peer-reviewed publications were excluded.

### Study Selection

On July 10, 2024, 2 researchers (CQZ and RL) conducted literature searches, reviewed the results, and imported them into Endnote X8 (Clarivate Analytics). CQZ was responsible for deduplication and the initial screening of studies, while RL reviewed CQZ’s selections. Both researchers then independently performed further screening based on the predefined inclusion criteria. Any disagreements were resolved through discussion between CQZ and RL. If a consensus could not be reached, a third researcher, GS, was consulted.

### Data Extraction

Data extraction was carried out by CQZ using a prespecified and tested form in Microsoft Excel. RL then reviewed the extracted data for accuracy. The information extracted included the first author, year of publication, age at follow-up, study design, study timeframe, country, data source, duration of follow-up, type of cancer reported, ART type (IVF, ICSI, FET, or fresh-ET), and case-control or exposure-nonexposure data. If any data were missing, the authors were contacted to obtain the necessary information.

### Quality Assessment and Risk of Bias

The Newcastle-Ottawa Quality Assessment Scale (NOS) was used for the quality assessment of the included studies [24]. Two authors (CQZ and RL) independently conducted the NOS evaluation, and any disagreements were resolved through discussions with the corresponding author or GS. Studies were categorized into low (total score ≥7), moderate (total score 5‐6), and high (total score ≤4) risk of bias.

Publication bias for the primary outcomes, such as ART versus non-ART and FET versus fresh-ET, was assessed using funnel plots and the Egger test. If the points on the funnel plot were symmetrically distributed, it indicated no or low bias; asymmetry suggested the presence of publication bias. The Egger test was performed to quantitatively assess publication bias, with a *P*<.05 indicating significant bias. Sensitivity analyses were conducted for the primary outcomes.

### Data Analysis and Synthesis

The RR and 95% CIs were chosen to assess the association between ART and childhood cancer in infertile offspring. Outcomes were combined using the DerSimonian and Laird random effects model [25]. All analyses were visualized using Stata 17 statistical software, and in meta-analyses, *P*<.05. Heterogeneity was analyzed using the *I*^2^ statistic. A high degree of heterogeneity was indicated if the *I*^2^ value was greater than 50%. Subgroup analyses were conducted based on the following four criteria: (1) continents, (2) duration of follow-up, (3) reported cancer type, and (4) operational versus nonoperational. Subgroup differences were assessed using the *Q* test, and statistical significance was defined as a *P*<.05. Regardless of the level of heterogeneity, a random-effects model was consistently applied to ensure the robustness of the analysis across different study designs and populations. Sensitivity analyses were performed by excluding each study individually.

### Quality of Evidence

GRADE (Grading of Recommendations Assessment, Development, and Evaluation) is a systematic approach for evaluating the quality of evidence by assessing 5 domains: methodological limitations (eg, risk of bias), heterogeneity of results (eg, inconsistency), generalizability of findings (eg, indirectness), precision of estimates, and risk of publication bias [26]. The overall certainty of the evidence is categorized into 4 levels, ranging from high to very low.

## Results

### Search Results and Study Characteristics

**Figure 1.**
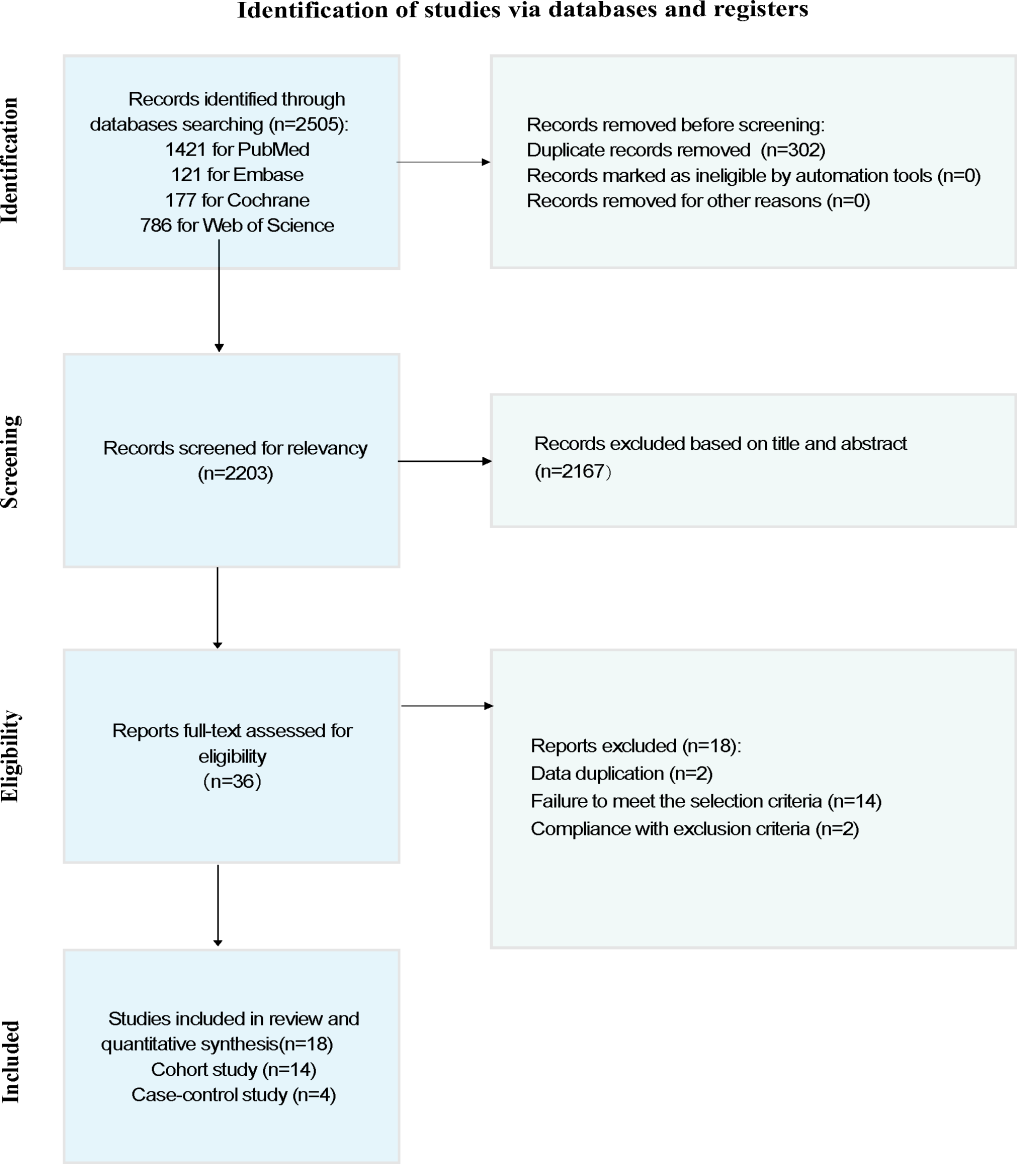
Flowchart illustrating the article selection process.

A total of 2505 articles were obtained from the systematic search, of which 302 (12.06%) were duplicates. We screened the titles and abstracts to exclude 2167 (86.51%) articles that did not meet the eligibility criteria and subsequently removed them. The full manuscripts of the 36 articles were screened to exclude 18 (50%) articles that did not meet the eligibility criteria. These included different papers by the same authors with duplicate data. Data that did not involve subfertile offspring were excluded. A total of 18 studies [15-22,27-36] were thus included in this review. The NOS quality of the included studies was either moderate or high (Table S1 in Multimedia Appendix 2) [15-22,27-36]. The PRISMA flowchart depicts the article screening process (Figure 1).

Of the 18 included studies, 14 (77%) were cohort studies [15-22,27-32] and 4 (22%) were case-control studies [33-36]. All cohort studies reported overall cancer occurrence risk, while the 4 case-control studies focused only on specific types of cancer, including retinoblastoma, leukemia, and neuroblastoma. All studies were published in English and covered multiple countries and regions, including Australia, Israel, Denmark, the United States, France, Finland, Sweden, the Netherlands, Taiwan, the United Kingdom, and Norway. The studies were published across nearly 2 decades, with the earliest study published in 2000 [27] and the most recent study published in 2024 [16]. Most cohort studies had follow-up durations ranging from 3 to 18 years, with the shortest follow-up period being 3 years [30] and the longest extending to 18 years [17].

Of the 18 studies, 10 (56%) [15-20,33-36] compared ART with non-ART and 11 (61%) [16,17,20-22,27-32] compared FET with fresh-ET. Of the 10 studies comparing ART with non-ART, 6 (60%) were cohort studies [15-20] involving 480,852 ART patients and 716,144 non-ART patients. Four (4/10, 40%) [33-36] were case-control studies involving 563 ART patients and 1521 non-ART patients. Of the 18 studies, 11 (61%) comparative studies of FET versus fresh-ET were cohort studies involving 176,800 FET patients and 723,327 fresh-ET patients.

### Comparison of Childhood Overall Cancer Risk by ART Conception and Non-ART Conception

**Figure 2.**
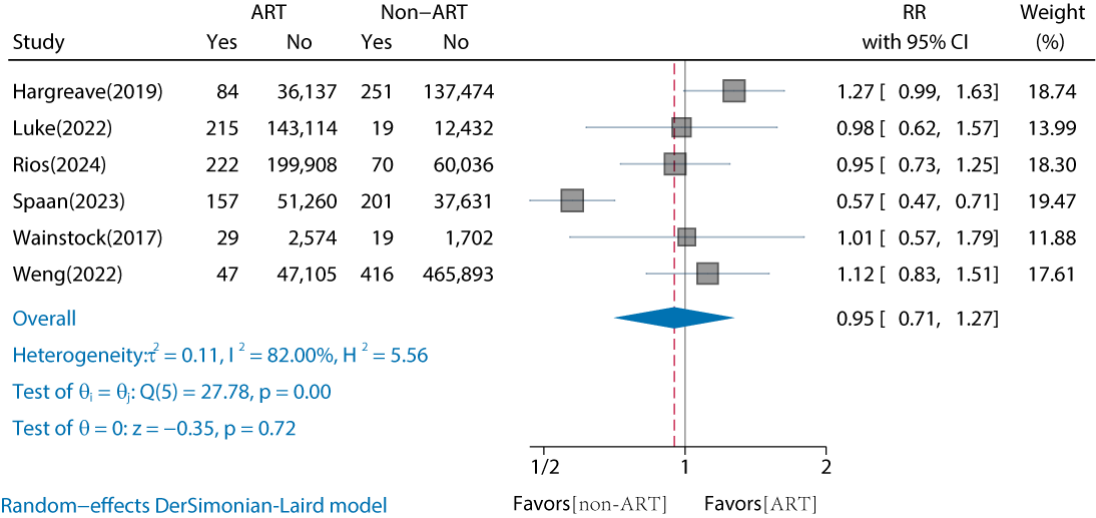
Comparison of childhood overall cancer risk by ART conception and non-ART conception [15-20]. ART: assisted reproductive technology; RR: relative risk.

Of the 18 studies, 6 (33%) studies have compared the risk of childhood overall cancer in offspring of ART versus non-ART conceptions (Table S2 in Multimedia Appendix 2) [15-20]. The results showed that there was no significant increase in the risk of childhood overall cancer in ART-conceived offspring compared with non-ART conception (RR 0.95, 95% CI 0.71‐1.27; *I*^2^=82%) (Figure 2). A high degree of heterogeneity was observed. We performed subgroup analyses based on continent, follow-up duration, reported cancer types, and whether artificial insemination procedures were involved. No significant differences were observed within the subgroups (Table 1). However, when the non-ART control group was defined as nonoperational (ie, using only OI or fertility drugs), the RR for childhood overall cancer in the ART group was 1.23 (95% CI 0.98‐1.54). Based on the GRADE evidence quality assessment, the quality of the comparison between ART and non-ART was rated as “low to moderate” due to serious risk of bias and inconsistency (Table S3 in Multimedia Appendix 2). The Egger test did not detect significant publication bias (*P*=.66), and the adjusted RR was 0.812 (95% CI 0.549‐1.074), indicating robust results (Figure S1 in Multimedia Appendix 3).

**Table 1.** Comparison of childhood overall cancer risk by ART^a^ conception and non-ART conception by subgroup analysis.

		Studies, n	ART, n	Non-ART, n	RR^b^ (95% CI)	*I*^2^ (%)	*P*_Heterogeneity_	*P*_Between groups_
Overall		6	480,852	716,144	0.95 (0.71‐1.27)	82	<.001	—^c^
Continents								.73
	Asian	2	49,755	468,030	1.09 (0.84‐1.43)	0	.76	—
	Europe	3	287,768	235,663	0.88 (0.54‐1.43)	91.83	<.001	—
	North America	1	143,114	12,451	0.98 (0.62‐1.57)	—	—	—
Duration of follow-up (years)								.69
	≤10	3	390,611	538,866	1.02 (0.84‐1.22)	0	.73	—
	>10	3	90,241	177,278	0.89 (0.49‐1.62)	91.61	<.001	—
Reported cancers type								.84
	Neoplasm	1	2603	1721	1.01 (0.57‐1.79)	—	—	—
	Overall cancer	5	478,249	714,423	0.94 (0.68‐1.31)	85.5	<.001	—
Operational versus nonoperational^d^								.10
	Non-ART (nonoperational)	2	38,711	139,176	1.23 (0.98‐1.54)	0	.47	—
	Non-ART (operational)	4	441,387	575,992	0.87 (0.61‐1.22)	82.08	<.001	—

a ART: assisted reproductive technology.

b RR: relative risk.

c Not applicable.

d We set non-ARTs that only use fertility drugs or ovulation induction as a nonoperational factor, and those that involve artificial insemination or intrauterine insemination operations as an operational factor.

In addition, we compared IVF, ICSI, FET, and fresh-ET conceptions with non-ART conceptions separately. The results showed no significant differences between either (*P*≥.05). The corresponding RRs were for IVF (RR 0.86, 95% CI 0.59‐1.25; *I*^2^=70.18%), ICSI (RR 0.76, 95% CI 0.26‐2.2; *I*^2^=94.61%), FET (RR 0.98, 95% CI 0.54‐1.76; *I*^2^=83.18%), and fresh-ET (RR 0.75, 95% CI 0.49‐1.15; *I*^2^=81.85%) (Figures S1-S4 in Multimedia Appendix 4) [15-18,20].

### Comparison of Childhood Overall Cancer Risk by FET Conception and Fresh-ET Conception

**Figure 3.**
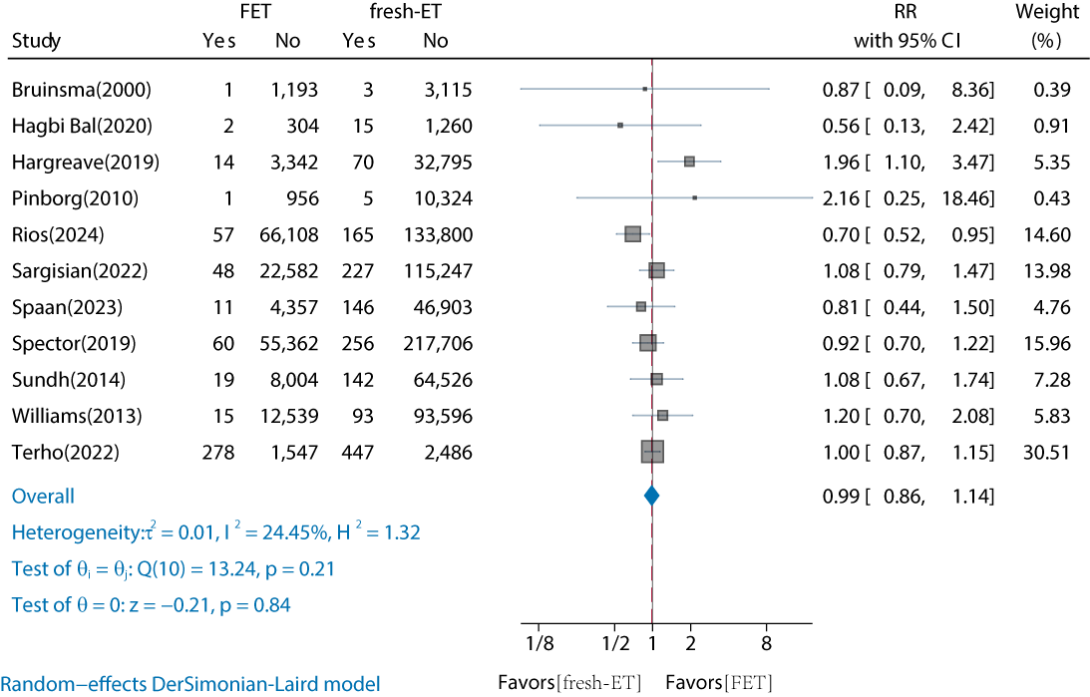
Comparison of childhood overall cancer risk by FET conception and fresh-ET conception [16,17,20-22,27-32]. FET: frozen embryo transfer; fresh-ET: fresh embryo transfer; RR: relative risk.

Of the 18 studies, 11 (61%) cohort studies compared the risk of childhood overall cancer in FET versus fresh-ET conceived offspring (Table S4 in Multimedia Appendix 2) [16,17,20-22,27-32]. The results showed no significant increase in the risk of childhood overall cancer for FET-conceived offspring compared to fresh-ET (RR 0.99, 95% CI 0.86‐1.14; Figure 3). The interstudy heterogeneity was low (*I*^2^=24.45%). Subgroup analyses by continent, follow-up duration, and cancer type revealed no significant differences (Table 2). Funnel plots and the Egger test indicated no publication bias (*t*=0.53, *P*=.61; adjusted RR 0.98, 95% CI 0.856‐1.125; Figure S2 in Multimedia Appendix 3). Based on the GRADE assessment, the quality of the comparison between FET and fresh-ET was rated as “moderate” due to a serious risk of bias (Table S3 in Multimedia Appendix 2).

**Table 2.** Comparison of childhood overall cancer risk by FET^a^ conception and fresh-ET^b^ conception by subgroup analysis.

		Studies, n	RR^c^ (95% CI)	*I*^2^ (%)	*P*_Heterogeneity_	*P*_Between groups_
Overall		11	0.99 (0.86‐1.14)	24.45	.21	—^d^
Continents						.21
	Asian	1	0.56 (0.13‐2.42)	—	—	—
	Europe	8	1.02 (0.84‐1.23)	43.58	.09	—
	North America	1	0.92 (0.7‐1.22)	—	—	—
	Oceania	1	0.87 (0.09‐8.36)	—	—	—
Duration of follow-up (years)						.37
	≤10	8	0.92 (0.79‐1.08)	0	.47	—
	>10	3	1.13 (0.75‐1.72)	64.27	.06	—
Reported cancers type						.94
	Neoplasm	2	0.99 (0.87‐1.14)	0	.44	—
	Overall cancer	9	1 (0.82‐1.23)	36.27	.13	—

a FET: frozen embryo transfer.

b fresh-ET: fresh embryo transfer.

c RR: relative risk.

d Not applicable.

### Comparison of Childhood-Specific Cancer Risk by ART Conception and Non-ART Conception

**Figure 4.**
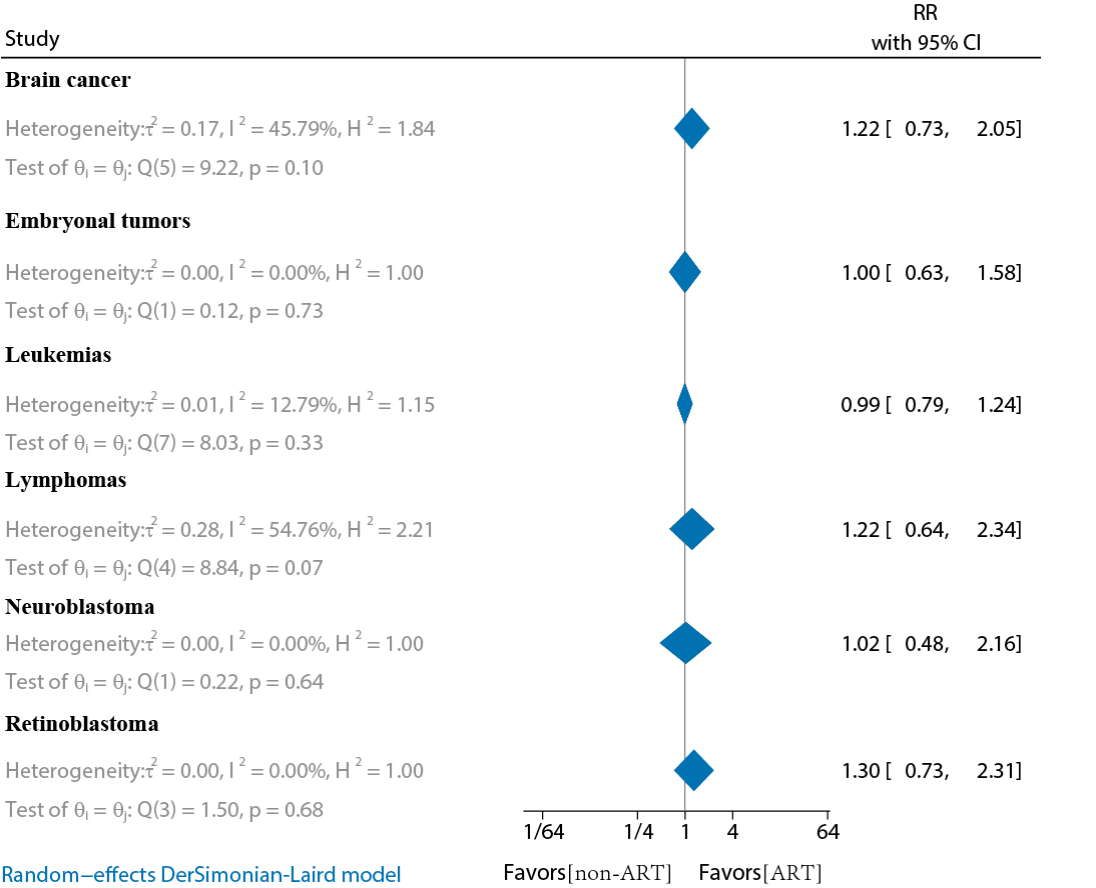
Comparison of childhood-specific cancer risk by ART conception and non-ART conception. ART: assisted reproductive technology; RR: relative risk.

In total, 10 studies compared the risk of childhood-specific cancer in the offspring of ART versus non-ART conceptions (Tables S5 and S6 in Multimedia Appendix 2) [15-20,33-36]. The main studies included 6 cohort studies and 4 case-control studies. The results showed that none of the ART-conceived offspring had a significantly increased risk of childhood-specific cancer compared to non-ART conception (*P*≥.05). The main ones included leukemia (RR 0.99, 95% CI 0.79‐1.24; *I*^2^=12.79%), lymphoma (RR 1.22, 95% CI 0.64‐2.34; *I*^2^=54.76%), brain cancer (RR 1.22, 95% CI 0.73‐2.05; *I*^2^=45.79%), embryonal tumors (RR 1, 95% CI 0.63‐1.58; *I*^2^=0%), retinoblastoma (RR 1.3, 95% CI 0.73‐2.31; *I*^2^=0%), and neuroblastoma (RR 1.02, 95% CI 0.48‐2.16; *I*^2^=0%) (Figure 4).

### Sensitivity Analysis

To explore the stability of the meta-analysis results, sensitivity analyses were performed by excluding each study individually. The results demonstrated the robustness of findings for both ART versus non-ART conception and FET versus fresh-ET conception regarding childhood cancer risk. The results remained consistent even after excluding the study by Spaan et al [17] (RR 1.1, 95% CI 0.95‐1.26; Figure S3 in Multimedia Appendix 3) [15-22,27-32].

## Discussion

### Principal Findings

This study, to our knowledge, is the first systematic review and meta-analysis to use infertile or subfertile populations as the reference group. We found no significant increase in childhood overall cancer risk in ART-conceived offspring compared to non-ART. This result was consistent across different ART methods, including IVF, ICSI, FET, and fresh-ET. Furthermore, no significant differences were observed between FET and fresh-ET in terms of childhood cancer risk. Despite the heterogeneity of the studies included, the results were robust across sensitivity analyses, supporting the stability and reliability of our findings.

### Comparison to Prior Work

To date, 6 meta-analyses have examined the association between ART fertility treatments and childhood cancer risk. The meta-analyses by Wang et al [12], Chiavarini et al [14], and Hargreave et al [10] found a significant correlation between ART and childhood cancer risk, while those by Raimondi et al [9], Gilboa et al [11], and Zhang et al [13] did not support such an association. Recent large-scale cohort studies have yet to reach a consensus on this issue. Some studies report a significant association between ART conception and increased childhood cancer risk [19,21,30,37,38]. For instance, a large Nordic study by Sargisian et al [21], which included data from Denmark, Norway, Sweden, and Finland, found a significantly increased risk of childhood cancer in ART-conceived offspring compared to naturally conceived offspring (RR 1.13, 95% CI 1.01‐1.26). However, other studies have not observed this association [16,17,20]. The inconsistencies in these findings may be due to differences in control group selection, sample size, and follow-up duration [5,6,8,39].

Recent evidence suggests that epigenetic changes may play a key role in causing infertility, rather than being simply a result of fertility treatments [40,41]. Couples experiencing infertility may already have a higher risk of epigenetic defects in their gametes, which fertility treatments have only helped to reveal [42]. The present meta-analysis provided new insights, and we selected an appropriate control group to eliminate the effects of infertile or subfertile. We included 6 large cohort studies involving 480,852 ART conceptions and 716,144 non-ART conceptions. In our analysis of the non-ART control group, we performed subgroup analysis by categorizing it into operational (IUI or artificial insemination) and nonoperational (OI or fertility drugs) groups. When the non-ART control group was defined as nonoperational, the RR for childhood overall cancer in the ART group increased to 1.23 (95% CI 0.98‐1.54), approaching the statistical significance threshold. The review by Berntsen et al [5] suggested that a scientific control group should consist of children of low-fertility parents who conceived naturally. However, obtaining such controls is challenging because they are rarely included in registry data. Additionally, it was noted that children born through fertility measures, such as ovarian stimulation or IUI, could also serve as suitable controls. Due to limitations in current published studies, we focused on studies with the latter control group approach. Future research should aim to include offspring born to low-fertility parents who conceived naturally to better understand the long-term effects of both infertility and fertility treatments.

ICSI has become increasingly common worldwide, with approximately one-third of fresh ART cycles using conventional IVF and two-thirds using ICSI [43,44]. Despite the invasive nature of ICSI and ongoing concerns about the health of children born through this method, our meta-analysis, which included 2 eligible studies, showed that the risk of childhood overall cancer in ICSI-conceived offspring was not significantly higher compared to non-ART offspring (RR 0.76, 95% CI 0.26‐2.2; *I*^2^=94.61%). However, there was considerable heterogeneity between the studies, and further research is needed to confirm the long-term safety of ICSI regarding childhood cancer risk.

FET accounts for 32.6% of all ART treatment cycles in Europe, showing a clear increasing trend [45]. Large cohort studies and meta-analyses have provided short-term health data on FET, such as perinatal outcomes [46-50]. Compared to singletons born after fresh-ET, infants born after FET generally have higher birth weights and a higher risk of LGA (large for gestational age) in suprapregnant children but lower perinatal mortality. Singletons born after FET are at an increased risk of LGA and preterm labor compared to naturally conceived offspring [50]. However, data on the long-term health of FET offspring are limited. Studies comparing FET with naturally conceived offspring suggest an increased cancer risk in FET-conceived children [20,21].

In our meta-analysis, which included 4 large cohort studies with infertile populations as the comparison group, we found no significant increase in childhood overall cancer risk in FET-conceived offspring compared to non-ART offspring (RR 0.98, 95% CI 0.54‐1.76; *I*^2^=83.18%). Given the high heterogeneity, these results should be interpreted with caution. Additionally, to our knowledge, no meta-analyses have compared childhood cancer risk between FET and fresh-ET conceived offspring. Our analysis, which included 11 cohort studies with 176,800 FET-conceived and 723,327 fresh-ET-conceived individuals, found no significant difference in cancer risk between the 2 groups (RR 0.99, 95% CI 0.86‐1.14), with low heterogeneity (*I*^2^=24.45%). No significant bias was found, and sensitivity analyses confirmed the stability of the results.

Several studies have explored the association between ART conception and specific childhood cancers, including leukemia [16,51], lymphoma [52], hepatoblastoma [31,53], retinoblastoma [54], and central nervous system tumors [15]. A 2013 meta-analysis by Hargreave et al [10] reported an increased risk of cancers such as leukemia (RR 1.65), neuroblastoma (RR 4.04), and retinoblastoma (RR 1.62). A 2019 meta-analysis by Chiavarini et al [14] found that ART significantly increased the risk of hematological neoplasms (odds ratio [OR] 1.3, 95% CI 1.08‐1.58) and neurological cancers (OR 1.21, 95% CI 1.01‐1.46). Furthermore, a 2020 study by Zhang et al [13] showed a significantly increased risk of hematologic cancers (RR 1.39), other solid tumors (RR 1.57), and leukemia (RR 1.31). Leukemia is one of the most common childhood cancers and a leading cause of death in children, followed closely by lymphoma and central nervous system tumors [55]. Although several studies have suggested that ART is associated with an increased risk of childhood leukemia, most compared ART offspring with those conceived naturally [16,19,20,30,56]. In contrast, our analysis included 8 studies on leukemia, 3 of which had follow-up durations of more than 10 years, and 2 were case-control studies. The results showed no significant increase in leukemia risk in ART offspring compared to non-ART offspring (RR 0.99, 95% CI 0.79‐1.24; *I*^2^=12.79%). When cohort studies were analyzed separately, the results remained unchanged (RR 1.1, 95% CI 0.87‐1.4; *I*^2^=4.05%). Additionally, no significant differences were found in further analyses of other specific childhood cancers, including lymphoma (RR 1.22, 95% CI 0.64‐2.34), brain cancer (RR 1.22, 95% CI 0.73‐2.05), embryonal tumors (RR 1, 95% CI 0.63‐1.58), retinoblastoma (RR 1.3, 95% CI 0.73‐2.31), and neuroblastoma (RR 1.02, 95% CI 0.48‐2.16).

### Strengths and Limitations

#### Strengths

One strength of this study is the use of a more appropriate control group, that is, infertile or subfertile populations, which enhances the reliability of the comparisons and helps address the risk of epigenetic defects associated with infertility. Additionally, our estimates were not significantly affected by recall bias, which is common in case-control studies [57]. Parents of children with cancer may be more likely to recall past events, potentially overestimating cancer risk. We combined 4 eligible case-control studies, mainly focusing on specific cancer types, and performed subgroup analyses, showing no significant differences across subgroups. Furthermore, large cohort studies with long-term and comprehensive data were recently included, reducing the risks of selection, attrition, and recall bias, while providing more opportunities to observe rare cancer exposures, thus enhancing the credibility of the findings.

#### Limitations

First, the sample size of infertile or subfertile populations was small. Despite a comprehensive search, the limited number of studies, especially on ICSI and FET offspring, may reduce confidence in the findings. Larger sample sizes are needed in future research for greater statistical power. Second, this meta-analysis did not classify the non-ART control group further. The lack of distinction between naturally conceived offspring from low-fertility parents and those conceived through ovarian stimulation or IUI may introduce confounding, affecting the cancer risk baseline. Future studies should differentiate these groups to better assess ART’s impact on childhood cancer risk. Third, while some studies reported male infertility, gender-specific analyses were not performed, preventing separate calculations for male and female infertility. Future studies should address this to explore gender-specific effects on offspring health after ART. Fourth, the included studies used raw data without adjusting for factors like age, gender, birth order, socioeconomic status, and history of abortion. This lack of adjustment may affect result interpretation. Future studies should include adjusted data for more accurate conclusions.

### Conclusions

This study found no significant difference in the risk of childhood cancer between offspring conceived through ART and those conceived through non-ART treatments (such as fertility drugs or OI/IUI) in infertile populations. While infertility treatments may elevate baseline risks, our findings suggest that whether individuals with infertility conceive using ART or non-ART methods, their offspring do not face a significantly higher risk of childhood cancer. Further research, especially comparing infertile populations who conceive naturally, is needed to better understand potential long-term health outcomes.

## Supplementary material

10.2196/65820 Multimedia Appendix 1 Literature search strategy.

10.2196/65820 Multimedia Appendix 2 Summary tables.

10.2196/65820 Multimedia Appendix 3 Funnel charts and sensitivity analyses.

10.2196/65820 Multimedia Appendix 4 Forest plot of cancer risk in offspring of different types of ART (IVF, ICSI, FET, and fresh-ET). ART: assisted reproductive technology; FET: frozen embryo transfer; fresh-ET: fresh embryo transfer; ICSI: intracytoplasmic sperm injection; IVF: in vitro fertilization.

10.2196/65820 Checklist 1 PRISMA (Preferred Reporting Items for Systematic Reviews and Meta-Analyses) checklist.

## Abbreviations

ART: assisted reproductive technology
FET: frozen embryo transfer
fresh-ET: fresh embryo transfer
GRADE: Grading of Recommendations Assessment, Development, and Evaluation
ICSI: intracytoplasmic sperm injection
INPLASY: International Platform of Registered Systematic Review and Meta-Analysis Protocols
IUI: intrauterine insemination
IVF: in vitro fertilization
LGA: large for gestational age
NOS: Newcastle-Ottawa Quality Assessment Scale
OI: ovulation induction
OR: odds ratio
PICOS: Population, Intervention, Comparison, Outcomes, and Study Design
PRISMA: Preferred Reporting Items for Systematic Reviews and Meta-Analyses
RR: relative risk
