# Supplementary material for: Assisted Reproductive Technology and Risk of Childhood Cancer Among the Offspring of Parents With Infertility: Systematic Review and Meta-Analysis
Source: JMIR Cancer. 2025 Mar 12;11:e65820. doi: 10.2196/65820 (PMC11921989; doi:10.2196/65820)
Supplement: Multimedia Appendix 1 [file cancer-v11-e65820-s001.doc]

**Multimedia Appendix 1**

**Literature search strategy.**

**Coverage: from the inception to 2024.07.10**

| **Database** | **Result** | **Search strings** |
| --- | --- | --- |
| PubMed | 1421 | (((Reproductive Techniques, Assisted[Mesh] OR assisted reproductive technology[Title/Abstract] OR ART[Title/Abstract] OR fertility treatment[Title/Abstract] OR in vitro fertilization[Title/Abstract] OR IVF[Title/Abstract] OR frozen embryo[Title/Abstract] OR ovarian stimulation[Title/Abstract] OR ICSI[Title/Abstract] OR intracytoplasmic sperm injection[Title/Abstract]) AND ((infant[MeSH] OR child[MeSH] OR adolescent[MeSH] OR pediatric[MeSH] OR paediatric[MeSH] OR children[MeSH] OR infant*[Title/Abstract] OR infancy[Title/Abstract] OR child[Title/Abstract] OR childhood[Title/Abstract] OR childhood pediatric[Title/Abstract] OR paediatric*[Title/Abstract] OR adolescen*[Title/Abstract] OR young[Title/Abstract] OR 18 kids[Title/Abstract] OR youth[Title/Abstract] OR juvenile[Title/Abstract] OR preschooler*[Title/Abstract] OR teen[Title/Abstract] OR teens[Title/Abstract] OR teenager*[Title/Abstract]))) AND (("Neoplasms/epidemiology"[Mesh] OR "Tumor*"[tiab] OR "cancer*"[tiab] OR "Neoplas*"[tiab] OR "carcino*"[tiab] OR "malignan*"[tiab] OR "onco*"[tiab] OR "hematoonco*"[tiab] OR Neuroblastoma OR Neuroectodermal Tumors, Primitive OR astrocytoma OR glioblastoma OR DIPG OR glioma OR medulloblastoma OR sarcoma OR osteosarcoma OR ewing OR ewings OR rhabdomyosarcoma OR wilms OR nephroblastoma OR retinoblastoma OR medulloblastoma OR teratoma OR germinoma OR dysgerminoma OR seminoma OR gonadoblastoma OR glioma OR carcinoma OR leukem* OR leukaem* OR lymphoma* OR leucocythaemia OR myelodysplastic syndrome OR myeloproliferative OR hodgkin disease OR HGG OR LGG OR ATRT OR PNET OR CML OR ALL OR AML OR JMML))) AND (("Risk"[Mesh] OR "Risk Assessment"[Mesh] OR "Risk Factors"[Mesh] OR "Odds Ratio"[Mesh] OR "Incidence"[Mesh] OR "Prevalence"[Mesh] OR "Probability"[Mesh:noexp] OR "Epidemiologic Studies"[Mesh] OR risk[ti] OR odds[ti] OR likelihood[ti] OR incidence[ti] OR prevalence[ti] OR propensit*[ti] OR probabilit*[ti] OR frequen*[ti] OR correlat*[ti] OR connect*[ti] OR epidemiolog*[ti] OR associat*[ti] OR relate*[ti] OR relationship[ti] OR "registries"[MeSH Terms])) |
| EMBASE | 121 | ('Reproductive Techniques, Assisted'/exp OR 'assisted reproductive technology':ab,ti OR 'ART':ab,ti OR 'fertility treatment':ab,ti OR 'in vitro fertilization':ab,ti OR 'IVF':ab,ti OR 'frozen embryo':ab,ti OR 'ovarian stimulation':ab,ti OR 'ICSI':ab,ti OR 'intracytoplasmic sperm injection':ab,ti) AND ('infant'/exp OR 'child'/exp OR 'adolescent'/exp OR 'pediatric'/exp OR 'paediatric'/exp OR 'children'/exp OR infant*:ab,ti OR infancy:ab,ti OR child:ab,ti OR childhood:ab,ti OR childhood pediatric:ab,ti OR paediatric*:ab,ti OR adolescen*:ab,ti OR young:ab,ti OR 18 kids:ab,ti OR youth:ab,ti OR juvenile:ab,ti OR preschooler*:ab,ti OR teen:ab,ti OR teens:ab,ti OR teenager*:ab,ti) AND ('Neoplasms/epidemiology'/exp OR 'Tumor*':ab,ti OR 'cancer*':ab,ti OR 'Neoplas*':ab,ti OR 'carcino*':ab,ti OR 'malignan*':ab,ti OR 'onco*':ab,ti OR 'hematoonco*':ab,ti OR 'Neuroblastoma':ab,ti OR 'Neuroectodermal Tumors, Primitive':ab,ti OR 'astrocytoma':ab,ti OR 'glioblastoma':ab,ti ) AND ('Risk'/exp OR 'Risk Assessment'/exp OR 'Risk Factors'/exp OR 'Odds Ratio'/exp OR 'Incidence'/exp OR 'Prevalence'/exp OR 'Probability'/exp OR 'Epidemiologic Studies'/exp OR risk:ab,ti OR odds:ab,ti OR likelihood:ab,ti OR incidence:ab,ti OR prevalence:ab,ti OR propensit*:ab,ti OR probabilit*:ab,ti OR frequen*:ab,ti OR correlat*:ab,ti OR connect*:ab,ti OR epidemiolog*:ab,ti OR associat*:ab,ti OR relate*:ab,ti OR relationship:ab,ti OR 'registries':ab,ti) |
| Cochrane Central Register of Controlled Trials | **177** | #1 MeSH descriptor: [Reproductive Techniques, Assisted] explode all trees  #2 ("assisted reproductive technology" or "ART" or "fertility treatment" or "in vitro fertilization" or "IVF" or "frozen embryo" or "ovarian stimulation" or "ICSI" or "intracytoplasmic sperm injection"):ti,ab,kw (Word variations have been searched)  **#3 #1 or #2**  #4 MeSH descriptor: [infant] explode all trees  #5 MeSH descriptor: [child] explode all trees  #6 MeSH descriptor: [adolescent] explode all trees  #7 MeSH descriptor: [pediatric] explode all trees  #8 ("infant" or "infancy" or "child" or "childhood" or "childhood pediatric" or "paediatric" or "adolescen" or "young" or "juvenile" or "teenager"):ti,ab,kw (Word variations have been searched)  **#9 #4 or #5 or #6 or #7 or #8**  #10 MeSH descriptor: [Neoplasms] explode all trees  #11 ("Tumor" or "cancer" or "Neoplas" or "carcino" or "malignan" or "onco" or "hematoonco" or "Neuroblastoma"):ti,ab,kw (Word variations have been searched)  **#12 #10 or #11**  #13 MeSH descriptor: [Risk] explode all trees  #14 MeSH descriptor: [Risk Assessment] explode all trees  #15 MeSH descriptor: [Risk Factors] explode all trees  #16 MeSH descriptor: [Odds Ratio] explode all trees  #17 MeSH descriptor: [Incidence] explode all trees  #18 MeSH descriptor: [Prevalence] explode all trees  #19 MeSH descriptor: [Probability] explode all trees  #20 MeSH descriptor: [Epidemiologic Studies] explode all trees  #21 ("risk" or "odds" or "likelihood" or "incidence" or "prevalence" or "propensit" or "relationship" or "registries"):ti,ab,kw (Word variations have been searched)  **#22 #13 or #14 or #15 or #16 or #17 or #18 or #19 or #20 or #21**  **#23 #3 and #9 and #12 and #22** |
| Web of Science | **786** | #1 TS=("Reproductive Techniques, Assisted" or "assisted reproductive technology" or "ART" or "fertility treatment" or "in vitro fertilization" or "IVF" or "frozen embryo" or "ovarian stimulation" or "ICSI" or "intracytoplasmic sperm injection")  #2 TS=("infant" or "infancy" or "child" or "childhood" or "childhood pediatric" or "paediatric" or "adolescen" or "young" or "juvenile" or "teenager")  #3 TS=("Neoplasms" or "Tumor" or "cancer" or "Neoplas" or "carcino" or "malignan" or "onco" or "hematoonco" or "Neuroblastoma")  #4 TS=("risk" or "Risk Assessment" or "odds" or "likelihood" or "incidence" or "prevalence" or "propensit" or "relationship" or "registries" or "Epidemiologic Studies")  #1 and #2 and #3 and #4 |
