# Supplementary material for: Assisted Reproductive Technology and Risk of Childhood Cancer Among the Offspring of Parents With Infertility: Systematic Review and Meta-Analysis
Source: JMIR Cancer. 2025 Mar 12;11:e65820. doi: 10.2196/65820 (PMC11921989; doi:10.2196/65820)
Supplement: Multimedia Appendix 2 [file cancer-v11-e65820-s002.doc]

**Multimedia Appendix 2**

**Summary tables**

**Table S1.** Characteristics of included studies.

| **Author** | **Year** | **Age at follow-up** | **Type of study** | **Study period** | **Country/region** | **Data sources** | **Impact of male infertility factors** | **Non-ART Group Subfertility Criteria** | **Follow-up(year, y)** | **Reported cancers** | **Quality scale^＃^** | **ART vs non-ART** | **FET vs ET** |
| --- | --- | --- | --- | --- | --- | --- | --- | --- | --- | --- | --- | --- | --- |
| Bruinsma [27] | 2000 | 0-15y | Cohort | 2004-2017 | Australia | NPSU | Some infertility individuals are attributed to male factors, but no independent analysis was conducted. | NA | 3y(median) | Overall cancer | 7 |  | ✔ |
| Hagbi Bal [28] | 2020 | 0-18y | Cohort | 1995-2018 | Israel | SUMC | Male/female infertility factors were not independently analyzed in the frozen vs. fresh embryo transfer comparison. | NA | 7.523y(median) | Neoplasms | 7 |  | ✔ |
| Hargreave [20] | 2019 | <20y | Cohort | 1996-2012 | Denmark | DIC | 1.Unspecified male infertility factors | 1、Undefined time of infertility  2、Non-ART Group = Fertility Drug Conceived Group | 11.3y (mean) | Overall cancer | 9 | ✔ | ✔ |
| Luke [15] | 2022 | NA | Cohort | 2004-2018 | US(Massachusetts, North Carolina, New York, and Texas) | SART CORS and state cancer and birth registries | 1.Male factors account for over one-third of infertility  2.No independent analysis of male factors | 1、Indicated by Infertility on Birth Certificate  2、Non-ART Group = OI/IUI Conceived Group | 7.2y(median) | Overall cancer | 9 | ✔ |  |
| Pinborg [29] | 2010 | 1-13y | Cohort | 1995-2007 | Denmark | national registers | 1.Unspecified male infertility factors | NA | 5.8y(mean) | Overall cancer | 7 |  | ✔ |
| Rios [16] | 2024 | / | Cohort | 2010-2021 | France | EPI-MERES | 1.Unspecified male infertility factors | 1、Undefined time of infertility  2、Non-ART Group = AI Conceived Group | 6.7y(median) | Overall cancer | 9 | ✔ | ✔ |
| Sargisian [21] | 2022 | 0-18y | Cohort | 1994-2014 | Denmark, Finland, Norway and Sweden | CoNARTaS (Committee of Nordic ART and Safety) | 1.Unspecified male infertility factors | NA | 9.9y(mean) | Overall cancer | 8 |  | ✔ |
| Spaan [17] | 2023 | NA | Cohort | 1983-2011 | Netherlands | 13 Dutch IVF clinics and two fertility centers;OMEGA-cohort | 1.Male infertility factor about 25%  2.No independent analysis of male factors | 1、time-to-pregnancy exceeding 12 months  2、Non-ART Group = NC/OI/IUI Conceived Group | 18y(median) | Overall cancer | 9 | ✔ | ✔ |
| Spector [30] | 2019 | 0-10y | Cohort | 2004-2012 | US | SART CORS and state cancer registries from 14 states. | 1.Unspecified male infertility factors | NA | 4.5y(mean) | Overall cancer | 8 |  | ✔ |
| Sundh [31] | 2014 | 0-15y | Cohort | 1982-2007 | Sweden, Denmark, and Norway | ESHRE | 1.Unspecified male infertility factors | NA | Average follow-up 9.5y | Overall cancer | 8 |  | ✔ |
| Wainstock [18] | 2017 | 0-18y | Cohort | 1991-2013 | Israel | SUMC | 1.Unspecified male infertility factors | 1、Undefined time of infertility  2、Non-ART Group = OI Conceived Group | 10.55y(median) | Total neoplasm | 7 | ✔ |  |
| Weng [19] | 2022 | 0-13y | Cohort | 2004-2017 | Taiwan | parents-child triads | 1.Male infertility factor about 6.26%  2.No independent analysis of male factors | 1、Undefined time of infertility  2、Non-ART Group = OI/IUI Conceived Group | 6y(median) | Overall cancer | 9 | ✔ |  |
| Williams [32] | 2013 | 0-15y | Cohort | 1992-2008 | UK | HFEA | Male/female infertility factors were not independently analyzed in the frozen vs. fresh embryo transfer comparison. | NA | 6.6y(mean) | Overall cancer | 8 |  | ✔ |
| Terho [22] | 2022 | NA | Cohort | 1995-2006 | Finland | FMBR | 1.Unspecified male infertility factors | NA | 18–20y(mean) | D00–D48, Neoplasms | 8 |  | ✔ |
| Foix-L’He´lias [33] | 2012 | 0-5y | Case-control | 2000-2006 | France | RNTSE | 1.Unspecified male infertility factors | 1、time-to-pregnancy exceeding 24 months  2、Non-ART Group = OI/IUI Conceived Group | NA | Retinoblastoma | 6 | ✔ |  |
| Rudant [34] | 2012 | 0-14 y | Case-control | 2003-2004 | France | structured telephone questionnaires; ESCALE study | 1.Unspecified male infertility factors | 1、time-to-pregnancy exceeding 12 months  2、Non-ART Group = OI/IUI Conceived Group | NA | Leukemia | 8 | ✔ |  |
| Ajrouche [35] | 2014 | ≤15y | Case-control | 2010-2011 | France | structured telephone questionnaires; ESTELLE study | The small sample size limited independent analysis. | 1、time-to-pregnancy exceeding 12 months/the need to consult a doctor  2、Non-ART Group = OI/IUI/Other treatments Conceived Group | NA | Leukemia | 7 | ✔ |  |
| Munzer [36] | 2007 | 0-15y | Case-control | 2003-2004 | France | structured telephone questionnaires; ESCALE study | 1.Unspecified male infertility factors | 1、Undefined time of infertility  2、Non-ART Group = OI/AI Conceived Group | NA | Neuroblastoma | 7 | ✔ |  |

Abbreviations: ART, Assisted Reproductive Technology; IVF, in vitro fertilization; ICSI, Intracytoplasmic Sperm Injection; FET, frozen-thawed embryo transfer; fresh-ET, Fresh embryo transfer; NC, natural conception; year, y; months, m; NA, not applicable; NPSU, clinics and the National Perinatal Statistics Unit; SUMC, Soroka University Medical Center; DIC, registry data and the Danish Infertility Cohort; SART CORS, Assisted Reproductive Technology Clinic Outcome Reporting System; EPI-MERES, French National Mother-Child Register; ESHRE, European Society for Human Reproduction and Embryology; HFEA,Human Fertilization and Embryology Authority; FMBR,Finnish Medical Birth Register; RNTSE, national reference centre for RB diagnosis and treatment;French national registry of children’s cancers.

^＃^Low (total score ≥ 7), moderate (total score 5-6), and high (total score ≤ 4) risk of bias.

**Table S2.** Included studies about the childhood overall cancer risk by ART conception and non-ART conception.

| **Author** | **Year** | **Continents** | **Follow-up(years)** | **Reported cancers** | **Operational vs. Non-Operational^*^** | **ART** | | | | | | **non-ART** | |
| --- | --- | --- | --- | --- | --- | --- | --- | --- | --- | --- | --- | --- | --- |
|  |  |  |  |  |  | **Total-ART** | **Cancer-ART** | **Total-IVF** | **Cancer-IVF** | **Total-ICSI** | **Cancer-ICSI** | **Total** | **Cancer** |
| Hargreave [20] | 2019 | Europe | ＞10 y | Overall cancer | Non-ART(non-operational group) | 36221 | 84 | 19448 | 38 | 13417 | 32 | 137725 | 251 |
| Luke [15] | 2022 | North America | ≤10 y | Overall cancer | Non-ART(operational group) | 143329 | 215 | NA | NA | NA | NA | 12451 | 19 |
| Rios [16] | 2024 | Europe | ≤10 y | Overall cancer | Non-ART(operational group) | 200130 | 222 | NA | NA | NA | NA | 60106 | 70 |
| Spaan [17] | 2023 | Europe | ＞10 y | Overall cancer | Non-ART(operational group) | 51417 | 157 | 33484 | 115 | 17933 | 42 | 37832 | 201 |
| Wainstock [18] | 2017 | Asian | ＞10 y | Neoplasm | Non-ART(non-operational group) | 2603 | 29 | 2603 | 29 | NA | NA | 1721 | 19 |
| Weng [19] | 2022 | Asian | ≤10 y | Overall cancer | Non-ART(operational group) | 47152 | 47 | NA | NA | NA | NA | 466309 | 416 |

Abbreviations: OI, Ovulation induction; AI,artificial insemination; ART, Assisted Reproductive Technology; IVF, in vitro fertilization; ICSI, Intracytoplasmic Sperm Injection (ICSI); NA, not applicable.

*We set non-ARTs that only use fertility drugs or Ovulation induction as a non-operational factor, and those that involve AI or IUI operations as an operational factor.

**Table S3.** The quality of meta-evidence based on GRADE scoring system.

| Exposure | n | Quality assessment | | | | | | | Effect | Quality |
| --- | --- | --- | --- | --- | --- | --- | --- | --- | --- | --- |
|  |  | Design | Risk of bias | Inconsistency | Indirectness | Imprecision | Publication bias | Increase grade consideration | 95% CI |  |
| ART vs non-ART | 6 | Cohort study | serious | Serious  (*I^2^*=82.00) | Not serious | serious* | Not serious | unsupported | RR 0.95  （0.71-1.27） | Low to moderate |
| FER vs fresh-ET | 11 | Cohort study | serious | Not serious (*I^2^*=24.45%) | Not serious | Serious^#^ | Not serious | unsupported | RR 0.99  （0.86-1.14） | moderate |

Note: n represents the number of included studies.

*Insufficient samples for some analyses, affecting statistical effectiveness

#Fewer cancer cases (especially in the FET subgroup)

**Table S4.** Included studies about the childhood overall cancer risk by FET conception and fresh-ET conception.

| Author | Year | Continents | Follow-up(years) | Reported cancers1 | FET | | Fresh-ET | |
| --- | --- | --- | --- | --- | --- | --- | --- | --- |
|  |  |  |  |  | Total | Cancer | Total | Cancer |
| Bruinsma [27] | 2000 | Oceania | ≤10 y | Overall cancer | 1194 | 1 | 3118 | 3 |
| Hagbi Bal [28] | 2020 | Asian | ≤10 y | Neoplasms | 306 | 2 | 1275 | 15 |
| Hargreave [20] | 2019 | Europe | ＞10 y | Overall cancer | 3356 | 14 | 32865 | 70 |
| Pinborg [29] | 2010 | Europe | ≤10 y | Overall cancer | 957 | 1 | 10329 | 5 |
| Rios [16] | 2024 | Europe | ≤10 y | Overall cancer | 66165 | 57 | 133965 | 165 |
| Sargisian [21] | 2022 | Europe | ≤10 y | Overall cancer | 22630 | 48 | 115474 | 227 |
| Spaan [17] | 2023 | Europe | ＞10 y | Overall cancer | 4368 | 11 | 47049 | 146 |
| Spector [30] | 2019 | North America | ≤10 y | Overall cancer | 55422 | 60 | 217962 | 256 |
| Sundh [31] | 2014 | Europe | ≤10 y | Overall cancer | 8023 | 19 | 64668 | 142 |
| Williams [32] | 2013 | Europe | ≤10 y | Overall cancer | 12554 | 15 | 93689 | 93 |
| Terho [22] | 2022 | Europe | ＞10 y | Neoplasms | 1825 | 278 | 2933 | 447 |

Abbreviations: FET, frozen-thawed embryo transfer; fresh-ET, Fresh embryo transfer.

**Table S5.** Included studies on childhood-specific cancer types.

| **Author** | **Year** | **Type of study** | **Reported cancers** | **ART-total** | **ART-Cancer** | **Non-ART-total** | **Non-ART-Cancer** |
| --- | --- | --- | --- | --- | --- | --- | --- |
| Hargreave [20] | 2019 | Cohort study | Leukemia | 37156 | 30 | 137725 | 80 |
|  |  |  | Lymphomas | 37156 | 8 | 137725 | 23 |
|  |  |  | Central nervous system neoplasms | 37156 | 25 | 137725 | 57 |
| Luke [15] | 2022 | Cohort study | Leukemia | 143329 | 57 | 12451 | 8 |
|  |  |  | CNS | 143329 | 49 | 12451 | 1 |
|  |  |  | Embryonal tumors | 143329 | 66 | 12451 | 5 |
| Rios [16] | 2024 | Cohort study | Leukemia | 200130 | 75 | 60106 | 19 |
|  |  |  | Lymphoma* | 133965 | 13 | 60106 | 6 |
|  |  |  | Malignant CNS tumor | 200130 | 43 | 60106 | 6 |
|  |  |  | Embryonal tumor | 200130 | 57 | 60106 | 18 |
| Spaan [17] | 2023 | Cohort study | Leukemia | 51417 | 31 | 37832 | 25 |
|  |  |  | Lymphoma | 51417 | 19 | 37832 | 23 |
|  |  |  | Brain | 51417 | 14 | 37832 | 14 |
|  |  |  | Retinoblastoma (C69) | 51417 | 7 | 37832 | 3 |
| Wainstock [18] | 2017 | Cohort study | Leukemia | 2603 | 0 | 1721 | 1 |
|  |  |  | Lymphoma | 2603 | 2 | 1721 | 0 |
|  |  |  | Brain | 2603 | 1 | 1721 | 1 |
|  |  |  | Ophthalmic | 2603 | 0 | 1721 | 1 |
| Weng [19] | 2022 | Cohort study | Leukemia | 47152 | 13 | 466309 | 104 |
|  |  |  | Lymphomas and reticuloendothelialneoplasms | 47152 | 5 | 466309 | 15 |
|  |  |  | CNS and miscellaneous intracranial and intraspinal neoplasms | 47152 | 5 | 466309 | 86 |
|  |  |  | Retinoblastoma | 47152 | 5 | 466309 | 33 |
|  |  |  | Neuroblastoma and other peripheralnervous cell tumors | 47152 | 5 | 466309 | 55 |
| Foix-L’He´lias [33] | 2012 | Case-control | Retinoblastoma | 496 | 7 | 1059 | 13 |
| Rudant [34] | 2012 | Case-control | Leukemia | 22 | 9 | 171 | 111 |
| Ajrouche [35] | 2014 | Case-control | Leukemia | 23 | 7 | 231 | 113 |
| Munzer [36] | 2007 | Case-control | All neuroblastoma | 22 | 3 | 60 | 6 |

*Missing data on fresh embryo transfer(fresh-ET)

**Table S6.** Comparison of childhood-specific cancer types risk by ART conception and non-ART conception.

| **Author** | **No. Of Studies** | **No. Of ART** | **RR (95% CI)** | ***I***^2^**(%)** | ***p***Heterogeneity | ***p***between groups |
| --- | --- | --- | --- | --- | --- | --- |
| Leukemia |  |  |  |  |  |  |
| Overall | 8 | 481832 | 0.99(0.79-1.24) | 12.79 | 0.33 |  |
| Type of study |  |  |  |  |  | 0.1 |
| Cohort study | 6 | 481787 | 1.10(0.87-1.40) | 4.05 | 0.39 |  |
| Case-control | 2 | 45 | 0.73(0.47-1.12) | 0 | 0.93 |  |
| **Lymphomas** |  |  |  |  |  |  |
| Overall | 5 | 272293 | 1.22(0.64-2.34) | 54.76 | 0.07 |  |
| **Brain cancer** |  |  |  |  |  |  |
| Overall | 6 | 481787 | 1.22(0.73-2.05) | 45.79 | 0.1 |  |
| **Retinoblastoma** |  |  |  |  |  |  |
| Overall | 4 | 101668 | 1.30(0.73-2.31) | 0 | 0.68 |  |
| Type of study |  |  |  |  |  | 0.74 |
| Cohort study | 3 | 101172 | 1.41(0.66-2.98) | 0 | 0.5 |  |
| Case-control | 1 | 496 | 1.15(0.46-2.86) | NA | NA |  |
| **Neuroblastoma** |  |  |  |  |  |  |
| Overall | 2 | 47174 | 1.02(0.48-2.16) | 0 | 0.64 |  |
| Type of study |  |  |  |  |  | 0.64 |
| Cohort study | 1 | 47152 | 1.32(0.36-4.88) | NA | NA |  |
| Case-control | 1 | 22 | 0.90(0.36-2.25) | NA | NA |  |
| **Embryonal tumour** |  |  |  |  |  |  |
| Overall | 2 | 343459 | 1.00(0.63-1.58) | 0 | 0.73 |  |

Abbreviations: Assisted Reproductive Technology (ART); RR, relative risk; NA, not applicable.
