# Supplementary material for: Assisted Reproductive Technology and Risk of Childhood Cancer Among the Offspring of Parents With Infertility: Systematic Review and Meta-Analysis
Source: JMIR Cancer. 2025 Mar 12;11:e65820. doi: 10.2196/65820 (PMC11921989; doi:10.2196/65820)
Supplement: Multimedia Appendix 3 [file cancer-v11-e65820-s003.doc]

**Multimedia Appendix 3**

**Funnel charts and sensitivity analyses**

**Figure S1.** Risk of publication bias assessment of comparison of childhood overall cancer risk by ART and non-ART conception.

Egger’s test: Test result: t = 0.48, p-value = 0.6537, Using the trim-and-fill method to address publication bias, the adjust RR was 0.812 (95% CI, 0.549 to 1.074).

**Figure S2.** Risk of publication bias assessment of comparison of childhood overall cancer risk by FET and fresh-ET conception.

Egger’s test: Test result: t = 0.53, p-value = 0.6094, Using the trim-and-fill method to address publication bias, the adjust RR was 0.98 (95% CI, 0.856 to 1.125).

**Figure S3.** Sensitivity analysis [15-22,27-32].

**A B**

A.comparison of any Childhood Cancer Risk by ART and Non-ART Conception.

B.comparison of any Childhood Cancer Risk by FET and fresh-ET Conception.
