# Supplementary material for: Assisted Reproductive Technology and Risk of Childhood Cancer Among the Offspring of Parents With Infertility: Systematic Review and Meta-Analysis
Source: JMIR Cancer. 2025 Mar 12;11:e65820. doi: 10.2196/65820 (PMC11921989; doi:10.2196/65820)
Supplement: Multimedia Appendix 4 [file cancer-v11-e65820-s004.doc]

**Multimedia Appendix 4**

**Forest plot of cancer risk in offspring of different types of ART (IVF, ICSI, FET, and fresh-ET)**

**Figure S1.** Comparison of any childhood overall cancer risk by IVF conception and non-ART conception [17,18,20].

Abbreviations: ART: assisted reproductive technology; IVF, in vitro fertilization; RR, relative risk; CI, confidence interval.

**Figure S2.** Comparison of any childhood overall cancer risk by ICSI conception and non-ART conception [17,20].

Abbreviations: ART: assisted reproductive technology; ICSI, intracytoplasmic sperm injection; RR, relative risk; CI, confidence interval.

**Figure S3.** Comparison of any childhood overall cancer risk by fresh-ET conception and non-ART conception [15-17].

Abbreviations: ART: assisted reproductive technology; fresh-ET, fresh embryo transfer; RR, relative risk; CI, confidence interval.

**Figure S4.** Comparison of any childhood overall cancer risk by FET conception and non-ART conception [15-17,20].

Abbreviations: ART: assisted reproductive technology; FET, frozen embryo transfer; RR, relative risk; CI, confidence interval.
